# Nitrate of Silver and Its Application in Diseased Conditions of the Mouth and Teeth

**Published:** 1894-11

**Authors:** J. M. Parker

**Affiliations:** Goldsboro, N. C.


ARTICLE IX.

### NITRATE OF SILVER AND ITS APPLICATION IN DISEASED CONDITIONS OF THE MOUTH AND TEETH.

J. M. PARKER, GOLDSBORO, N. C.

Nitrate of silver is conceded to rank as one of the most efficient and reliable remedies in medicine and surgery, and when its merits are fully known, we believe that it will be found equally efficient in a large class of diseases of the teeth.

A saturated solution of nitrate of silver I have used with much satisfaction on diseased pulps, since the beginning of my practice of dentistry. Especially well does it act on exposed nerves of temporary teeth when divitalization is necessary. It does not penetrate them as does carbolic acid, nor does it involve the entire pulp in an inflammatory process, as does arsenious acid.

Sometimes you could not well use the solution, because of inability to dry the cavity sufficiently to let the remedy do its work. Then the powdered crystals can be used with advantage.

With a child suffering with sensitive dentine with teeth inclined to ache, where there seems to be some abrasion, nothing acts so well as a solution applied on the surface after having been dried with chloroform and alcohol. Blow hot air over this surface, thereby making the nitrate of silver sink into the tubuli of the dentine. Flow over this dry surface oxiphosphate. To illustrate this take a strong solution of nitrate of silver and put a drop on a piece of hard or glazed paper, and you will see when evaporation of water takes place and how much of the silver is left, and how it seems to adhere to the smooth surface.

In badly decayed and broken down teeth and roots with dead nerves or live nerves, nothing is more sure to act as a preserver to the seeming disintegrated gum margins and roots of teeth than the strong solution of nitrate of silver. Here I first use chloroform and alcohol to drive off all moisture and take out as far as possible, all infiltrated impurities in the dentine, and then apply the nitrate solution and again dry and fill as you see fit, or as the case demands.

When a child's teeth are to be treated, and it cannot be kept quiet long enough to dry the mouth, I use heated gutta-percha, first, bringing it to touch with powdered nitrate of silver, or, if this cannot be made to stick use cotton slightly filled with sandarac varnish with nitrate of silver on this. In a week this can be removed, if it is still in place, and a more permanent application made. In three Months time, on removal of this, to put in permanent filling, the cavity will be found black and hard, and with no sensation on being cut.

When extreme sensitiveness about the necks of the teeth, at the margin of the gums, where the tendency is to softening of the tissues of the tooth, a condition very annoying to the patient, and troublesome to the dentist, nitrate of silver has proved more successful with me than any other remedy in checking the progress of the disease and relieving the patient. It may be applied to the sensitive part without pain to the patient. The use of the strong solution is best here, because it will adhere to the parts, and after it is dried, soft oxiphosphate can be flowed over it to hold it in place and prevent contact with the soft tissues. In erosion, or wasting of the teeth, the strong solution is applied to the affected parts, and after being dried covered with cement till it is firmly established in the dentine.

Where the progress of the disease has gone so far as to require restoration by filling, this preliminary treatment is beneficial in preventing a further waste of the tooth substance and consequent failure of the operation. In superficial decay in soft teeth, where dark surfaces are not objectionable, nitrate of silver is admirable. By removing the softened portion of the tooth, polishing the surface and applying the saturated solution, giving it time to dry in and varnishing its parts to protect till taken into the organic structure, you will have a dense, hard surface free from sensitiveness. In filling cavities, having an excess of organic matter, an application of a four per cent solution of nitrate of silver, after having the cavity bathed with absolute alcohol, will effectually prevent after-sensitiveness. This treatment will result, at all times, in a darkish hue to the walls of the filling. This I explain to the patients, that they may know that it results from the treatment, and it is a proper and favorable condition for permanency of the operation. Also, in crowns and bridges where the dentine is uncovered, it is beneficial to use this remedy on the teeth and roots, used to sustain the crown or bridge as a protection against chemical changes or decay.

In badly broken down teeth with cavities extending below the gum, and when it is impossible for us to use the rubber dam and the matrix, and a leakage of the surrounding tissues is liable to enter the cavity while introducing the filling and injure the permanency of the operation, cauterize with nitrate of silver.

In pyorrhea, after the deposits from the roots of the teeth are removed, a very strong solution applied on cotton has proved more beneficial than any remedy I have tried. Gently pressing the cotton between the teeth and allowing it to remain a few minutes is sufficient, and then washing the parts with water by syringe.

Sometimes after extirpation of pulps we find the root canals very sensitive, at or near the apex especially. A strong solution of the nitrate of silver, say just enough water to dissolve the crystal, carried to the roots and hot air applied and then left sealed up in the roots for several hours, usually relieves the trouble and the cavity can be filled without pain or danger of abscess.

By utilizing this simple, though honest remedy, with the forces of nature, combined with common sense and perseverance, we may accomplish much. And when we have been successful with any remedy, if it is only to assist in changing a broken down and seemingly worthless member of the "dental family" into an honest and peaceful citizen, we have done much.—*Southern Dental Journal.*

